# Dapagliflozin plus exenatide on patients with type 2 diabetes awaiting bariatric surgery in the DEXBASU study

**DOI:** 10.1038/s41598-022-07250-z

**Published:** 2022-02-25

**Authors:** Carolina López-Cano, Maria Dolores Santos, Enric Sánchez, Raquel Martí, Marta Bueno, Liliana Gutiérrez-Carrasquilla, Albert Lecube

**Affiliations:** 1 Endocrinology and Nutrition Department, University Hospital Arnau de Vilanova de Lleida, Obesity, Diabetes and Metabolism Research Group (ODIM), Institut de Recerca Biomèdica de Lleida (IRBLleida), University of Lleida, Avda. Rovira Roure 80, 25198 Lleida, Spain; 2 Centro de Investigación Biomédica en Red de Diabetes y Enfermedades Metabólicas Asociadas (CIBERDEM), Instituto de Salud Carlos III (ISCIII), Madrid, Spain

**Keywords:** Bone, Bone imaging

## Abstract

The glucagon-like peptide-1 receptor agonist family together with the renal sodium/glucose cotransporter-2 inhibitors have garnered interest as potential therapeutic agents for subjects with type 2 diabetes and obesity. In these patients, bariatric surgery is indicated based in a BMI ≥ 35 kg/m^2^. A 24-week non-blinded, randomized pilot study to assess the efficacy of subcutaneous exenatide 2.0 mg once weekly plus oral dapagliflozin 10 mg once daily (Group A) compared to a control group (Group B) in 56 patients with type 2 diabetes awaiting bariatric surgery was conducted (EudraCTid.: 2017-001,454-33). Both groups received an energy-deficit low-fat diet. The primary endpoint was the proportion of patients running off the criteria for bariatric surgery at the end of the follow-up period (BMI ≤ 35.0 kg/m^2^ or a BMI ≤ 40.0 kg/m^2^ plus an HbA1c ≤ 6.0%). Changes in the BMI were also of interest. The proportion of patients who ran off the criteria for bariatric surgery was larger in Group A than in the control group (45.8% *vs.* 12.0%, p = 0.010). Participants in Group A exhibited an absolute decrease in body weight and BMI of 8.1 kg (95%IC: − 11.0 to − 5.2) and 3.3 kg/m^2^ (95%IC: − 4.5 to − 2.2), respectively (p < 0.001 for both in comparison with Group B). A higher percentage of participants in Group A reached a BMI < 35 kg/m^2^ (45.8 vs 12.0%) and lost > 10% of their initial body weight (20.8 vs 0%) compared to Group B. The combination of exenatide plus dapagliflozin appears as a strategic option to reduce the waiting list for bariatric surgery, especially in those patients with type 2 diabetes.

## Introduction

The prevalence of obesity has increased dramatically in recent decades and is more than 30% in some European countries^1^. Obesity increases the risk of a variety of comorbid conditions, including hypertension, dyslipidaemia, obstructive sleep apnoea and type 2 diabetes^2^. Since type 2 diabetes is associated with obesity, and obesity is the main etiological cause of type 2 diabetes, the term ‘diabesity’ has been proposed^3^. Unfortunately, few safe and effective drugs are currently available for the treatment of obesity, leading to a marked increase in use of surgery to treat it in Western countries^4^. However, less than 2% of patients with severe obesity potentially eligible for bariatric surgery eventually undergo these procedures^5^. Therefore, a massive residual pool of patients with diabetes that meet medical guidelines to undergo bariatric surgery is still waiting for a solution.

In recent years, there has been an increased understanding of the role of gastrointestinal hormones, such as the glucagon-like peptide-1 (GLP-1), in the control of glucose metabolism and body weight^6,7^. In the human pancreas, actions of GLP-1 include the enhancement of glucose-dependent insulin synthesis and secretion, and stimulation of beta-cell proliferation^7^. In addition, GLP-1 also plays a role in regulating satiety, feeding behaviour, and body weight^6^. As compared with healthy controls, patients with type 2 diabetes and obesity have an attenuated secretion of GLP-1 in response to either an oral carbohydrated load or a mixed meal^8,9^. In this setting, the GLP-1 receptor agonist (GLP-1ra) family has garnered intense interest as a potential therapeutic agent for subjects with diabesity^6,7,10^.

More recently, the emergence of inhibitors of the renal sodium-linked glucose transporter type 2 (SGLT2i) has provided more hope to patients with diabesity^11,12^. The SGLT2, located mainly in the first two segments of the proximal tubular system, mediates most of the renal reabsorption of glucose from the glomerular filtrate. Therefore, its therapeutic inhibition induces glucose excretion with urine, reaching two beneficial effects for patients with type 2 diabetes: glycaemic control and weight loss^13,14^.

On this basis, our purpose was to evaluate the percentage of patients with obesity and type 2 diabetes who no longer met the criteria for bariatric procedures after a combined therapy with both GLP-1ra plus SGLT2i added to an hypocaloric diet. Therefore, we designed a non-blinded, randomized, pilot study in patients with type 2 diabetes awaiting for bariatric surgery to assess the efficacy of subcutaneous exenatide 2.0 mg once weekly plus oral dapagliflozin 10 mg once daily, together with a low-fat, energy-deficient diet. The control group only received an energy-deficit low-fat diet.

## Results

No differences at baseline were observed between both groups regarding the main anthropometry, clinical and metabolic parameters, nor in the measurement of quality of life. (Table 1).

**Table 1:** Anthropometric, clinical and metabolic characteristics at baseline of the 56 participants in the DEXBASU study.

	ALL POPULATION	GROUP A	GROUP B	*p*
n	56	26	30	–
Age (yrs)	50.4 ± 7.5	49.1 ± 8.2	51.5 ± 6.7	0.255
Women, n (%)	20 (37.0)	8 (32.0)	12 (41.3)	0.335
Caucasian, n (%)	50 (92.5)	23 (92.0)	27 (93.1)	0.505
BMI (kg/m^2^)	38.4 ± 2.5	38.4 ± 2.4	38.4 ± 2.6	0.957
Waist circumference (cm)	122.8 ± 8.6	122.3 ± 8.2	123.2 ± 9.1	0.730
Hypertension, n (%)	32 (57.1)	14 (53.8)	18 (60.0)	0.783
Systolic BP (mmHg)	142.8 ± 17.5	143.6 ± 18.6	142.1 ± 16.8	0.751
Diastolic BP (mmHg)	81.8 ± 10.1	81.3 ± 10.8	82.2 ± 9.7	0.744
FPG (mg/dl)	191.9 ± 64.2	190.2 ± 67.0	193.4 ± 62.6	0.859
HbA1c (%)	8.3 ± 0.8	8.4 ± 0.7	8.2 ± 0.8	0.595
HbA1c (mmol/mol)	67.7 ± 8.9	68.4 ± 8.3	67.7 ± 9.5	0.595
Dyslipidemia, n (%)	40 (71.4)	21 (80.7)	19 (63.3)	0.212
c-LDL (mg/dl)	113.9 ± 34.5	120.2 ± 37.5	108.5 ± 31.4	0.221
c-HDL (mg/dl)	41.8 ± 8.8	40.8 ± 11.0	42.6 ± 6.4	0.448
Triglycerides (mg/dl)	200.0 (126.0 to 273.0)	270.0 (172.5 to 323.5)	149.0 (114.5 to 214.0)	0.004
Cardiovascular disease, n (%)	5 (8.9)	3 (11.5)	2 (6.6)	0.653
IWQOL-Lite	65.4 ± 25.6	63.2 ± 26.2	67.3 ± 25.4	0.570

Data are mean ± SD, median (interquartile range) or n (percentage). Group A: exenatide 2.0 mg once weekly plus oral dapagliflozin 10 mg once daily along with a low-fat diet about 500 kcal per day deficit; Group B; a low-fat diet with about 500 kcal per day deficit; BMI: body mass index; BP: blood pressure; FPG: fasting plasma glucose; HbA1c: glycated haemoglobin; c-LDL: low density lipoproteins cholesterol; c-HDL: high density lipoproteins cholesterol; IWQOL-Lite: Impact of Weight on Quality of Life-Lite. The prevalence of dyslipidemia was obtained from patients who during the study period had a diagnostic code for disorders of lipoprotein metabolism according to of the International Classification of Diseases codes. The incidence of blood hypertension was obtained from subjects who had an identification code for hypertensive diseases.

**Figure 1:**
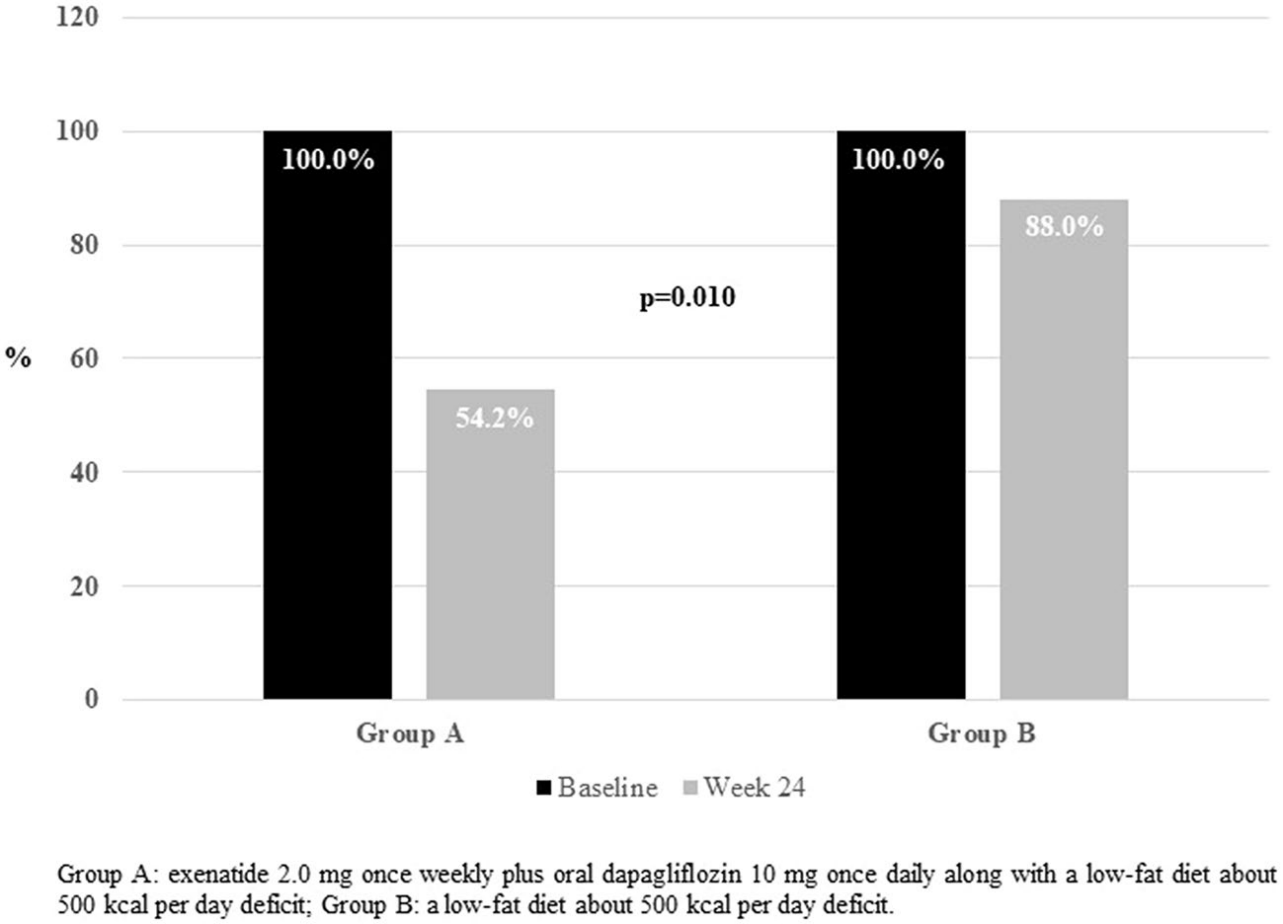
Proportion of patients accomplishing the criteria for bariatric surgery at baseline and after the 24-week follow-up period in both groups in the DEXBASU study.

After a follow-up period of 24 weeks, the proportion of patients running off the criteria for bariatric surgery was significantly higher in patients under treatment with dapagliflozin plus exenatide (Group A) in comparison with the control group (Group B) (45.8% *vs.* 12.0%, p = 0.010) (Fig. 1). In addition, when patients who still met surgical criteria at the end of 24 weeks were asked if they wanted to continue with the surgical protocol, 23.0% in Group A and 50.0% in Group B answered “yes” (p = 0.112).

**Figure 2:**
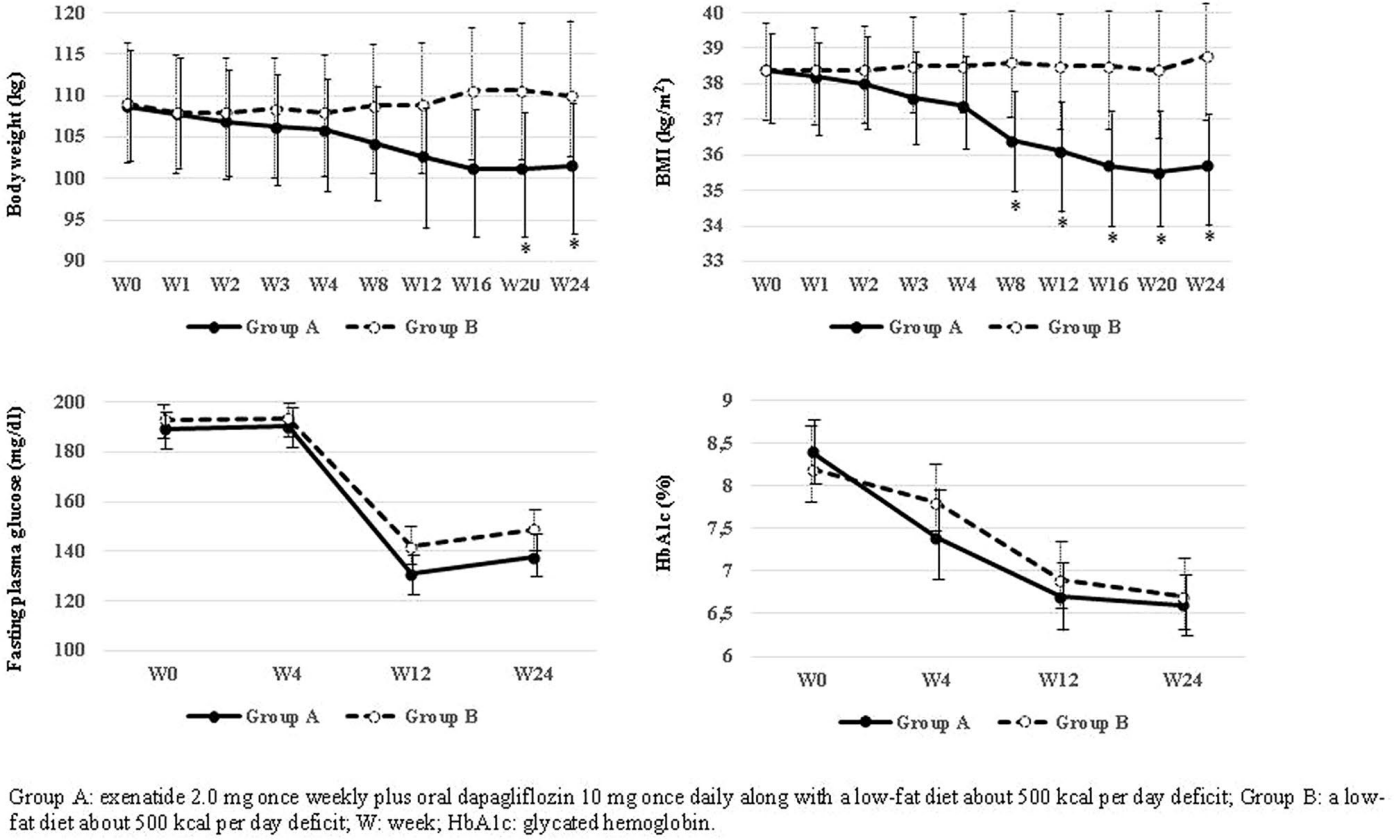
Evolution of weight, BMI, fasting plasma glucose and glycated hemoglobin from baseline to week 24 in the DEXBASU study.

In parallel, participants in Group A exhibited an absolute decrease in their body weight and BMI of 8.1 kg (95%IC: − 11.0 to − 5.2) and 3.3 kg/m^2^ (95%IC: − 4.5 to − 2.2), respectively, in comparison with participants in Group B (p < 0.001 for both) (Table 2). The decline in weight and BMI was progressive along the 24-weeks follow-up period, with differences in BMI becoming evident from week 8 (Fig. 2).

**Table 2:** Evolution from baseline to 24 weeks of anthropometric, clinical and metabolic data of participants in the DEXBASU study according to the received treatment, together with analysis of treatment effect.

		BASAL	24 WEEKS	MEAN DIFFERENCE (95% CI)	*p*
BMI (kg/m^2^)	Group A	38.5 ± 2.5	35.7 ± 3.1	− 2.7 (− 3.6 to − 1.9)	< 0.001
	Group B	38.2 ± 2.7	38.8 ± 3.4	0.6 (− 0.2 to 1.4)	0.133
	Δ	–	–	− 3.3 (− 4.5 to − 2.2)	< 0.001
Weight (kg)	Group A	108.2 ± 13.1	101.6 ± 15.5	− 6.6 (− 8.5 to − 4.6)	< 0.001
	Group B	108.4 ± 14.8	110.0 ± 17.0	1.5 (− 0.6 to 3.7)	0.162
	Δ	–	–	− 8.1 (− 11.0 to − 5.2)	< 0.001
Waist circumference (cm)	Group A	122.2 ± 8.3	118.6 ± 9.0	− 3.6 (− 6.0 to − 1.1)	0.005
	Group B	122.6 ± 9.0	123.4 ± 9.6	0.8 (− 1.6 to 3.3)	0.492
	Δ	−	−	− 4.4 (− 7.8 to − 1.0)	0.011
FPG (mg/dl)	Group A	189.2 ± 68.5	137.6 ± 34.8	− 51.6 (− 80.8 to − 22.4)	0.001
	Group B	193.0 ± 67.1	148.9 ± 49.7	− 44.0 (− 70.0 to − 18.0)	0.002
	Δ	–	–	− 7.6 (− 45.6 to 30.3)	0.688
HbA1c (%)	Group A	8.3 ± 0.7	6.6 ± 0.8	− 1.6 (− 2.0 to − 1.3)	< 0.001
	Group B	8.2 ± 0.8	6.7 ± 0.7	− 1.5 (− 1.9 to − 1.0)	< 0.001
	Δ	–	–	− 0.1 (− 0.7 to 0.4)	0.535
HbA1c (mmol/mol)	Group A	67.9 ± 8.2	49.4 ± 9.2	− 18.5 (− 22.6 to − 14.3)	< 0.001
	Group B	66.3 ± 9.7	49.9 ± 7.8	− 16.4 (− 21.5 to − 11.2)	< 0.001
	Δ	−	−	− 2.1 (− 8.0 to − 4.6)	0.535
Systolic BP (mmHg)	Group A	144.0 ± 18.9	130.8 ± 18.9	− 13.2 (− 20.3 to − 6.1)	0.001
	Group B	143.3 ± 17.3	136.1 ± 21.3	− 7.2 (− 15.6 to 1.2)	0.092
	Δ	–	–	− 6.0 (− 16.8 to 4.7)	0.269
Diastolic BP (mmHg)	Group A	81.4 ± 11.0	80.2 ± 11.5	− 1.1 (− 6.0 to 3.6)	0.623
	Group B	82.6 ± 10.3	77.8 ± 11.7	− 4.8 (− 9.3 to − 0.2)	0.040
	Δ	−	−	3.6 (− 2.8 to 10.0)	0.264
c-HDL (mg/dl)	Group A	41.0 ± 11.2	42.2 ± 10.6	1.2 (− 0.8 to 3.2)	0.238
	Group B	43.0 ± 6.8	45.2 ± 8.9	2.2 (− 0.1 to 4.6)	0.066
	Δ	–	–	− 1.0 (− 4.1 to 2.0)	0.505
Triglycerides (mg/dl)	Group A	270.0 (172.5 to 323.5)	214.5 (149.5 to 236.5)	–	0.063
	Group B	149.0 (114.5 to 214.0)	160.0 (101.0 to 221.0)	–	0.476
	Δ	–	–	− 31.0 (− 66.5 to 34.5)	0.112
c-LDL (mg/dl)	Group A	121.7 ± 37.5	106.0 ± 43.5	− 15.7 (− 27.0 to − 4.3)	0.009
	Group B	110.9 ± 32.1	110.7 ± 33.6	− 0.2 (− 12.5 to 12.1)	0.972
	Δ	–	–	− 15.5 (− 31.9 to 0.8)	0.063
IWQOL-Lite total score	Group A	63.7 ± 27.4	58.5 ± 26.9	− 5.1 (− 13.8 to 3.5)	0.229
	Group B	66.7 ± 25.9	64.0 ± 29.7	− 2.6 (− 8.0 to 2.6)	0.313
	Δ	–	–	− 2.5 (4.8 to − 12.1)	0.605

Data are mean ± SD or median (interquartile range). Group A: exenatide 2.0 mg once weekly plus oral dapagliflozin 10 mg once daily along with a low-fat diet about 500 kcal per day deficit; Group B; a low-fat diet with about 500 kcal per day deficit; BMI: body mass index; FPG: fasting plasma glucose; HbA1c: glycated haemoglobin; BP: blood pressure; c-LDL: low density lipoproteins cholesterol; c-HDL: high density lipoproteins cholesterol; IWQOL-Lite: Impact of Weight on Quality of Life-Lite.

**Figure 3:**
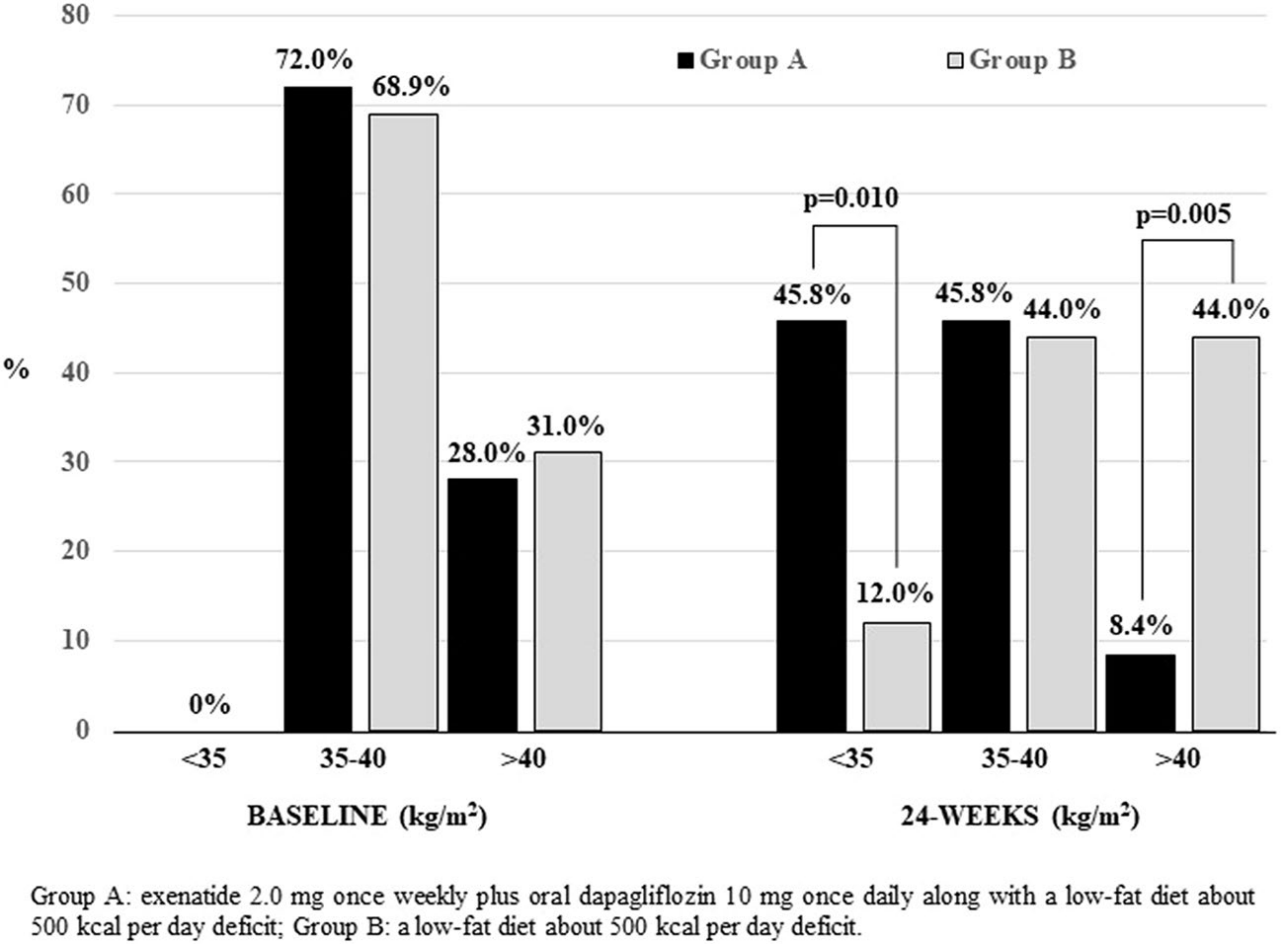
Evolution of body weight categories according to BMI from baseline to 24-weeks in the DEXBASU study.

Waist circumference also experienced a significant decrease among participants receiving dapaglifloxin plus exenatide in comparison with the control group [− 4.4 (− 7.8 to − 1.0) cm, p = 0.011] (Table 2). Regarding classifications in BMI categories, at the end of the 24-weeks period, a 45.8% of participants in Group A achieved a BMI < 35 kg/m^2^ in comparison with the 12.0% of participants in Group B (Fig. 3). On the other hand, the proportion of participants with a BMI > 40.0 kg/m^2^ was significantly lower among participants in Group A than participants in Group B (8.4% *vs*. 44.0%, p = 0.005). Similarly, the percentage of participants who lost both > 5% (58.3% *vs*. 16.0%, p = 0.002) or > 10% (20.8 *vs.* 0%, *p* = 0.022) of their initial body weight were higher in Group A than in Group B. Similar results were detected for changes in anthropometric measurements when men and women were assessed separately.

Contrary to weight changes, the evolution of glucose metabolism was comparable between groups, with a significant decrease from baseline to week 24 in fasting plasma glucose and HbA1c (Table 2, Fig. 2). The proportion of patients who achieved a decrease of 1.0% in their baseline HbA1c was also correlated: 87.5% in Group A and 68.0% in Group B (*p* = 0.097). However, the composite objective of > 5.0% decrease in body weight plus a > 1.0% decrease in HbA1c from baseline to 24-weeks of therapy was achieved in the 50% of patients under exenatide plus dapagliflozine in comparison with the 16.0% in Group B (p = 0.012). There was not a significant effect treatment in lipid profile, in blood pressure or in the IWQOL-Lite (Table 2).

Eleven patients in Group A and 10 patients in Group B reported a total of 19 and 10 adverse events (AE), respectively. Seven of the 29 AEs were related to gastrointestinal disorders (15.7% in Group A vs. 30.0% in Group B). In Group A, 4 AEs related to administration site conditions (21.0%) and 3 related to urinary tract infections (15.7%) were reported, with no cases in Group B. No participants in either treatment group experienced confirmed hypoglycaemia or hypotension, and neither AE caused treatment discontinuation. One patient in Group B experienced twice the same serious adverse event (a non-fatal heart failure episode that required inpatient hospitalization and was resolved with persisting sequales).

## Discussion

To the best of our knowledge, the DEXBASU study is the first to provide clinical evidence that the combination of exenatide plus dapagliflozin exhibits a positive impact on patients with type 2 diabetes awaiting for bariatric surgery. This combination achieves a reduction by almost half the number of patients who meet the criteria for bariatric surgery through its double effect on weight and hyperglycemia. Considering that both severe obesity and type 2 diabetes will increase their prevalence in the coming years, and when bariatric surgery is accessible to a very limited number of patients, combinations of pharmacological treatments emerge as a valid option for both patients as for the National Health Systems^15,16^.

The disappointing medium and long-term results in body weight provided by dietary and behavioral treatment, as well as the dearth of drug treatment, have led to an exponential increase in use of surgery to treat obesity in Western countries^4^. In fact, bariatric surgery is an effective therapy for obesity with an acceptable safety profile and provides appropriate treatment for people who do not achieve the recommended treatment goals with medical therapies, especially when there are other important comorbidities such as type 2 diabetes ^5^. The *International Federation for the Surgery of Obesity and Metabolic Diseases* reported that almost 580,000 metabolic procedures were performed worldwide in 2014^17^. Similarly, the *American Society for Metabolic and Bariatric Surgery* estimated that in the United States the initially 158.000 metabolic procedures performed in 2011 raised to 252.000 in 2018^18^. However, although this exponential increase in obesity-related surgical procedures less than 2% of eligible patients are treated annually^4,5^. Determining factors to explain these data include lack of awareness of the surgical option in some communities and negative attitudes towards obesity, but also misunderstanding about optimal management of patients with obesity and type 2 diabetes and the role of metabolic surgery procedures in its treatment scheme. According to the 1991 *National Institutes of Health* guidelines patients with type 2 diabetes appears as candidates for metabolic surgery if their BMI is higher or equal to 35 kg/m^2^^19^. This group of patients with type 2 diabetes mellitus and candidates for surgery have been the study population in the DEXBASU study.

Both GLP-1ra and SGLT2i, with complementary mechanisms of action, have been shown to be very useful options in the treatment of type 2 diabetes, particularly in patients with obesity^20,21^. Treatment-associated weight loss can be significant, with a low risk of hypoglycemia, and some molecules provide proven cardiovascular benefits, which differentiates these classes of drugs from most other antidiabetic agents^22^. Therefore, the combined treatment of GLP-1ra plus SGLT2i could be an attractive option for patients with type 2 diabetes and obesity awaiting bariatric surgery. However, no previous data was previously available in this particular setting.

The DURATION-8 study recruited 695 patients (47.9% of men) with type 2 diabetes poorly controlled with metformin, with a mean BMI of 32.7 kg/m^2^^23^. For 52 weeks the combination of exenatide once weekly plus dapagliflozin once daily showed greater average body weight loss than exenatide and dapagliflozin alone, with 37.7% of participants achieving an HbA1c < 7.0% and a 30.7% of participants who achieved weight loss ≥ 5.0% at the end of the follow-up period. These benefits had already been achieved by reaching week 28 and extended to 104 weeks after randomization^24,25^. In combination therapy participants, reductions in body weight were greater in women than in men, in patients with basal HbA1c ≥ 8.0% to < 9.0%, and as BMI increased^26^. In the DEXBASU study, in which all participants had a BMI ≥ 35 kg/m^2^, the percentage of subjects who managed to lose > 5% of their baseline body weight increased to 58.3%. In addition, no differences in the evolution of anthropometric measurements between men and women were observed in our study. Weight reduction with this drug combination is associated with a decresase in total volume, abdominal visceral and subcutaneous adipose tissue assessed by magnetic resonance imaging of body composition, and also with improved markers of liver steatosis such as fatty liver index and FIB-4 scores^27–29^. Combination therapy with exenatide and dapagliflozin may also have synergistic effects on markers of renal function compared with therapy alone or with placebo in obese patients with type 2 diabetes^30^. In an attempt to explain the possible beneficial effects of the combination of exenatide and dapagliflozin in poorly controlled type 2 diabetes, Ferrannini et al.suggested that the combination abolished the dapagliflozin-induced rise in the β-hydroxybutyrate, reduced the exenatide induced increase in fasting insulin-to-glucagon molar ratio, maintained glycosuria, and increased haematocrit, while mitigating the risk of ketoacidosis^31^.

In patients with obesity without type 2 diabetes, increased body weight loss after 24 weeks with exenatide plus dapagliflozin was associated with lower basal adiposity and lower basal insulin secretion, as well as with the single-nucleotide polymorphisms rs10010131, a variant associated with the GLP-1 pathway^32^. Unfortunately, we do not have data regarding the body composition of participants in the DEXBASU study.

Different combinations, beyond exenatide and dapagliflozin, have been evaluated in randomized clinical trials. In the AWARD-10 dulaglutide was assesed as adjunctive therapy to SGLT2i in patients with inadequately controled type 2 diabetes^33^. Again, the result was a significant and clinically relevant improvement in glycaemic control and weight loss with dulaglutide 1.5 mg once weekly compared to placebo after a 24-week follow-up period. Similarly, in the SUSTAIN-9 trial, when 1.0 mg subcutaneous semaglutide was added once weekly to SGLT2i therapies for 30 weeks, HbA1c [− 1.42% (95% CI: − 1.61 to − 1.24)] and body weight [− 3.81 kg (− 4.70 to − 2.93)] were further reduced versus those randomised to volume-matched placebo^34^. Lastly, the efficacy and safety of insulin degludec/liraglutide (IDegLira) was evaluated when added-on to SGLT2i therapy in patients with inadequately controlled type 2 diabetes. IDegLira did not show inferiority with respect to insulin glargine 100 units/mL regarding to the average reduction in HbA1c, but superiority with body weight [− 1.92 kg (− 2.64 to − 1.19)]^35^. In two recent meta-analysis, the GLP-1ra/SGLT2i combination was associated with an improved HbA1c control, along with enhanced weight reduction and control of blood pressure and lipid profile^36,37^. In the DEXBASU study, both groups achieved a similar reduction in HbA1c, which must be attributed to the intensification of glycemic control in both groups. The fact that the percentage of patients treated with pioglitzone (a peroxisome proliferator-activated gamma receptor agonist that is highly effective in the treatment of insulin resistance but is associated with adverse events such as weight gain and retention of liquids) in group B increased from 8.0% at the beginning to 60.0% at the end of the follow-up could help to better understand the differences in the evolution of weight.

Although age is a limitation for bariatric surgery, Carretero Gómez et al.evaluated the real-world efficacy and safety of SGLT2i and GLP-1ra combination therapy in 113 patients with type 2 diabetes over 65 years with an average BMI of 36.5 kg/m^2^^38^. After 6 months of follow-up, a significant reduction in HbA1c levels (− 1.1%), BMI (− 2.1 kg/m^2^) and systolic blood pressure (− 13 mmHg) was observed. In addition, the greatest reduction in HbA1c levels and weight was observed in patients who started both drugs simultaneously.

There are a some potential limitations that need to be considered when contemplating the results of our study. First, we evaluated a pretty minor number of patients with type 2 diabetes and obesity who were inclined to participate in a clinical trial, meaning that no irrefutable clinical consequences can be inferred to general population. Second, the treatment period was limited to 24 weeks, when we know that obesity is a chronic and recurrent condition. Therefore, longer-term studies are needed in the same population. Third, it was a pilot study with a control group lacking a placebo, which may weaken the power of our findings. Fourth, we cannot rule out that changes in the treatment of comorbidities, including diabetes, may have influenced part of the results.

In conclusion, given the opportunity to achieve remarkable reductions in body weight and ameliorate glycemic control, without further risk of hypoglycemia, our data strongly support the GLP-1ra/SGLT2i combination as a strategic option in the management of patients with type 2 diabetes awaiting bariatric surgery. By providing a rational basis for designing new strategies for the treatment of patients with obesity and type 2 diabetes, the information obtained from the DEXBASU study may be useful in reducing the healthcare costs associated with diabesity and will contribute towards improving the quality of life of these patients.

## Methods

### Statement on ethics and reporting philosophy

The DEXBASU study was approved by the *Spanish Agency for Medicines and Health Products* as well as for the human ethics committee of the Arnau de Vilanova University Hospital (CEIC 2017/0801). All potential participants provisioned a written informed consent to join the trial, which was conducted according to the Helsinki Declaration and the Good Clinical Practice Guidelines. The DEXBASU trial has been registered in the European Clinical Trial Database (EudraCT identifier: 2017–001,454-33; date of registration: 22/06/2017). The reporting has been done following the CONSORT guideline for randomized trials^39^.

### Study design and participants

**Figure 4:**
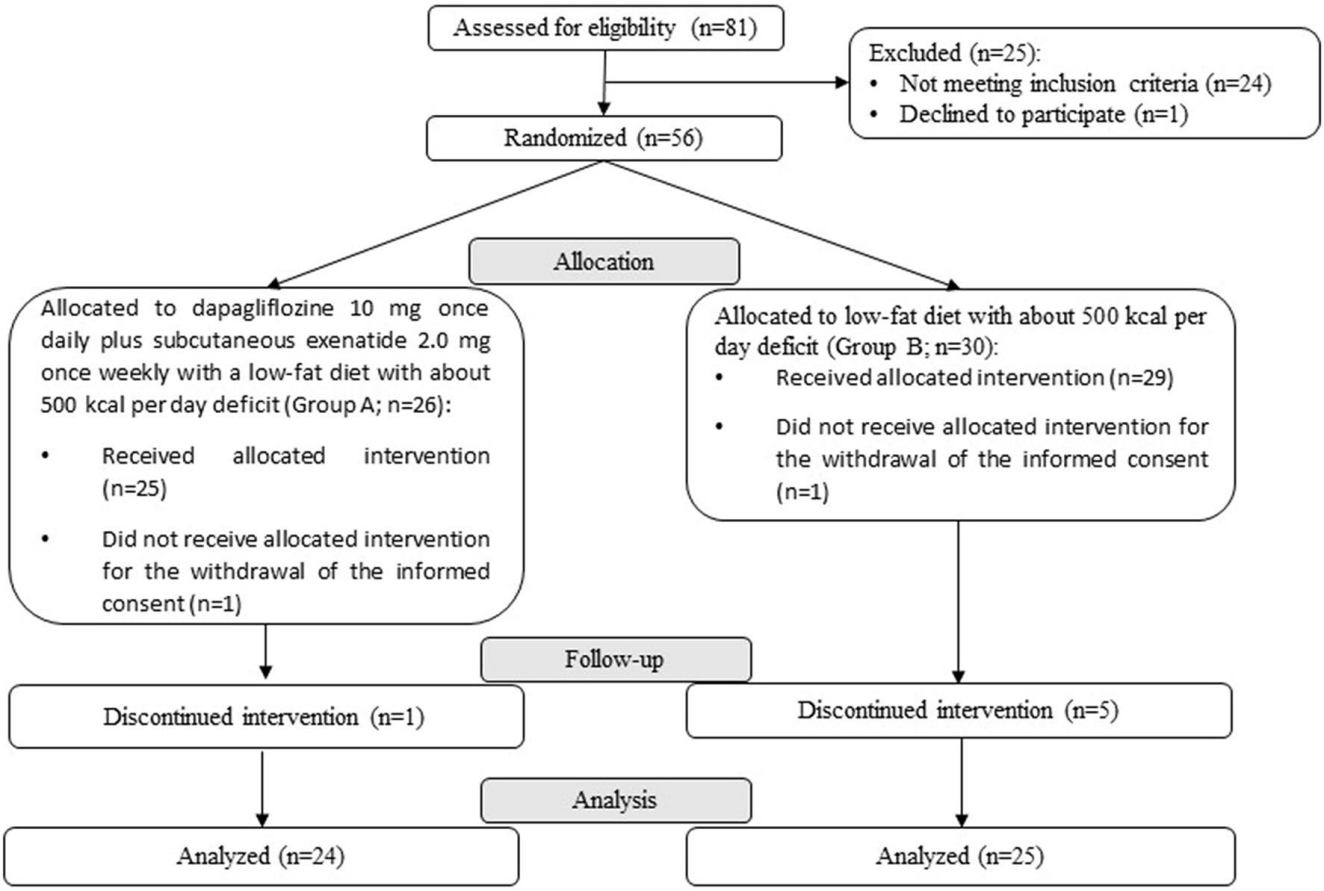
CONSORT 2010 Flow Diagram for the DEXBASU study.

The DEXBASU study is a pilot, phase II, randomized, non-blinded study to assess the efficacy of subcutaneous exenatide 2.0 mg once weekly plus dapagliflozin 10 mg once daily in a 24 weeks duration trial in patients with type 2 diabetes awaiting bariatric surgery. The study was performed in the Arnau de Vilanova University Hospital of Lleida. This center is one of the *Centers of Obesity Management* under the umbrella of the *European Association for the Study of Obesity* (COM-EASO). The study was open for recruitment from November 2017 to May 2020, when the target sample size was obtained. The study flow chart is displayed in Fig. 4.

Eligible participants included men and women aged 18–60 years, with type 2 diabetes and a body mass index (BMI) between 35.0 to 42.5 kg/m^2^ that were awaiting bariatric surgery. This implies a time of evolution of severe obesity greater than 5 years. The cut-off of 42.5 kg/m2 was an arbitrary set point. Other inclusion criteria comprised stable body weight (< 5% reported change during the previous 3 months) and HbA1c between 7.0 and 10.0%.

Exclusion criteria included previous surgical obesity procedures, use of approved weight lowering pharmacotherapy, previous or active treatment with GLP-1ra, SGLT2i or warfarin, current drug or alcohol abuse, uncontrolled psychiatric illness, an estimated Glomerular Filtration Rate < 60 ml/min/1.73m^2^, serum creatinine > 1.7 mg/dl, abnormal liver function test (alanine aminotransferase and aspartate aminotransferase levels greater than three times the upper limit of normal), pregnancy, breast-feeding and females of childbearing potential who were not using adequate contraceptive methods. Patients were also excluded from the trial if there was a history of acute or chronic pancreatitis, cholelithiasis, personal or familial history of medullary thyroid carcinoma or multiple endocrine neoplasia type 2, cardiovascular disease, heart failure and/or stroke, or recurrent urinary tract or genital infections. Patients enrolled in the study who initiated SGLT2i, GLP-1ar or oral corticosteroids were also excluded from the final analysis.

### Sample size calculation

This was a pilot study in which we assumed that a GLP-1ra plus SGLT2i based therapy will lead to an absolute 30% risk increase of the rate of patients with type 2 diabetes and obesity running off the *National Institutes of Health* criteria for bariatric surgery comparing with placebo at the end of the 24-week follow-up period^19^. Therefore, the inclusion of 46 patients achieved a 80% of statistical power (β = 0.2), with a significant level of 5% (α = 0.05) to detect this significant increase. However, sample size was rised to a total of 56 patients according to an expected drop-out rate of 20% in relation to adverse drug effects.

### Inclusion phase, treatment groups and titration

After signing informed consent and verify compliance with all the inclusion and none of exclusion criteria, patients were randomly allocated in a 1:1 ratio to one of the two treatment groups. Randomisation was done using a permuted block design with a computer random number generator by the Contract Research Organisation. In addition, individuals were stratified according to sex and BMI category (< 35.0, 35 to 39.9 and ≥ 40.0 kg/m^2^). Participants assigned to Group A received: **(i)** 2-week initial period with dapagliflozin 10 mg once daily, along with an energy-deficit low-fat diet, followed by **(ii)** 22-week of dapagliflozin 10 mg once daily plus subcutaneous exenatide 2.0 mg once weekly, along with a reduction in their daily energy intake to 500 kcal below their individual requirements. Participants assigned to Group B underwent a 24-week treatment period with a low-fat diet with about 500 kcal per day deficit below their individualized requirements, that were assessed using prediction equations. Qualified professionals prescribed the calorie reduction diet in person on day 0, and the advice was repeated at each visit throughout the study. No specific recommendations on increasing physical activity were given to the participants. All participants started the assigned treatment within 2 weeks of randomization. Twenty four participants from Group A and 25 participants from Group B completed the treatment period of 24 weeks (Fig. 4). A post-trial safety follow-up visit 4 to 6 weeks after the trial completion was performed in all participants.

### Type 2 diabetes treatment at baseline and during the follow-up period

At baseline, patients were treated with metformin (91.6 and 92.0%), sulphonylureas (25.0 and 20.0%), dipeptidyl peptidase-4 inhibitors (50.0 and 44.0%), pioglitazone (0.0 and 8.0%), basal insulin (33.3 and 32.0%), and fast-acting insulin (4.1 and 0.0%) in Groups A and B, respectively. None patient was treated with diet alone.

All subjects underwent treatment intensification to improve glycemic control according to our routine medical practice, except for the exclusion of SGLT2i and GLP-1ar. At the end of the study, the proportion of patients receiving metformin (91.6 and 92.0%), and fast-acting insulin (4.1 and 4.0%) in Groups A and B was almost the same. However, modifications were observed in those treated with sulphonylureas (16.0 and 36.0%), dipeptidyl peptidase-4 inhibitors (0.0 and 80.0%), pioglitazone (0.0 and 60.0%), and basal insulin (16.6 and 36.0%), respectively.

### Outcome measures

The primary endpoint was to assess the proportion of patients with type 2 diabetes awaiting bariatric surgery running off the *National Institutes of Health* criteria for bariatric surgery after the 24-week follow-up period ^16^. Mainly, proportion of patients who achieved a BMI ≤ 35.0 kg/m^2^ or a BMI ≤ 40.0 kg/m^2^ plus an HbA1c ≤ 6.0%.

As secondary endpoints the study assessed the effects of treatment from baseline to 24-week on: **(i)** body weight, classification of weight category by BMI (< 35 kg/m^2^, 35–40 kg/m^2^, and > 40 kg/m^2^) and waist circumference; **(ii)** percentage of patients who achieve both a 5% and 10% decrease in body weight; **(iii)** percentage of patients who achieve a decrease of 1% in their HbA1c level; **(iv)** proportion of patients who achieve the composite objective of both a ≥ 5% decrease in body weight plus a ≥ 1.0% decrease in HbA1c levels; **(v)** changes in blood pressure and lipid profile; and **(vi)** changes in quality of life and and proportion of patients that refuse the surgery option at the end of the follow-uop period.

Weight and height were measured without shoes and light clothing with and standard equipment, to the nearest 0.5 kg and 1.0 cm, respectively. BMI was calculated as the ratio of body weight (kg) to the height (m) squared ^40^. Waist circumference was measured with a non-stretchable tape with an accuracy of 0.1 cm, midway between the lowest rib and the iliac crest, on the horizontal plane with the subject in a standing position^41^. All measurements were performed by one experienced nurse, using the same device to avoid inter-observer and inter-device variability. The *Impact of Weight on Quality of Life-Lite* (IWQOL-Lite) was administered to quantitatively assess the individual’s perception of how weight affects day-to-day life^42^. It is comprised of 31 items grouped into five dimensions: physical functioning, self-esteem, sexual life, public distress, and work. The measure provides scores for each separate dimension and a total score.

### Statistical analysis

A normal distribution of the variables was established using the Kolmogorov–Smirnov test, and data are expressed as the mean ± standard deviation, median (interquartile range), or as a frequency (percentage). A homogeneity analysis was undergone to compare patients in both groups with regards to basal clinical and anthropometric variables. Mann–Whitney test for categorical variables and Student’s *t* test for continuous variables were used to assess differences between groups. All endpoints were analyzed longitudinally from randomization to 24 weeks and cross-sectional at 24 weeks. A chi-squared or exact Fisher test was done to compare the proportion of patients who don’t accomplish the *National Institutes of Health criteria* for bariatric surgery at the end of the study. All the contrasts were bilateral with a threshold for statistical significance threshold set at 0.05 (p < 0.05). The data was analyzed with the Statistical Package for the Social Sciences software (IBM SPSS Statistics for Windows, Version 20.0. Armonk, NY, USA).

Baseline description of the study population was made in the intention to treat population (all randomized). Efficacy analyzes were performed on the per protocol population (restricted to participants who completed the study without major protocol deviations), whereas safety analyzes were carried out on the safety population (participants who received at least one dose of any study product).
